# A 6-month, multicenter, open-label study of fixed dose naproxen/esomeprazole in adolescent patients with juvenile idiopathic arthritis

**DOI:** 10.1186/s12969-018-0260-y

**Published:** 2018-06-26

**Authors:** Daniel J. Lovell, Jason A. Dare, Megan Francis-Sedlak, Julie Ball, Brian D. LaMoreaux, Emily Von Scheven, Adam Reinhardt, Rita Jerath, Oral Alpan, Ramesh Gupta, Donald Goldsmith, Andrew Zeft, Henry Naddaf, Beth Gottlieb, Lawrence Jung, Robert J. Holt

**Affiliations:** 1 Cincinnati Children’s Hospital Medical Center, 3333 Burnet Ave, Cincinnati, OH 45229 USA; 2 University of Cincinnati School of Medicine, 3230 Eden Ave, Cincinnati, OH 45267 USA; 3 Arkansas Children’s Hospital, 1 Children’s Way, Slot# 512-2, Little Rock, AR 72202 USA; 4 Horizon Pharma USA, Inc, 150 South Saunders Road, Lake Forest, IL 60045 USA; 5 University of California San Francisco Pediatric Rheumatology, 550 16th Street, 5th Fl, San Francisco, CA 94158 USA; 6 University of Nebraska Medical Center/Children’s Hospital and Medical Center, 8200 Dodge St, Omaha, NE 68114 USA; 7 Augusta University Medical Center, 1120 15th Street, Augusta, GA 30912-5536 USA; 8 O & O Alpan, LLC, 11212 Waples Mill Rd Ste. 100, Fairfax, VA 22030 USA; 9 Rheumatology and Immunology Private Practice, 6005 Park Ave, Suite 409, Memphis, TN 38119 USA; 10 St. Christopher’s Hospital for Children, 160 E Erie Ave, Philadelphia, PA 19134 USA; 11 The Cleveland Clinic, 9500 Euclid Avenue, Cleveland, OH 44195 USA; 12 Toledo Clinic Inc, 4235 Secor Road, Toledo, OH 43623 USA; 13 Cohen Children’s Medical Center of New York, 269-01 76th Avenue, New Hyde Park, NY 11040 USA; 14 Children’s National Medical Center, 111 Michigan Avenue, NW, Washington, DC, 20010 USA; 15 Department of Pharmacy Practice, College of Pharmacy, University of Illinois-Chicago, 1721 North Woods Way, Vernon Hills, IL 60061 USA

**Keywords:** Juvenile idiopathic arthritis, Non-steroidal anti-inflammatory drugs (NSAIDs), Naproxen, Esomeprazole

## Abstract

**Background:**

Juvenile idiopathic arthritis (JIA) is an inflammatory arthritis of unknown etiology, which lasts for greater than 6 weeks with onset before 16 years of age. JIA is the most common chronic rheumatic disease in children. NSAIDs have been the mainstay of initial management with naproxen (NAP) being commonly used, but they may cause serious side effects such as gastric ulcers which can be reduced by concomitant administration of proton pump inhibitors, such as esomeprazole (ESO).

**Methods:**

Primary objective was to evaluate the safety and tolerability of 3 fixed doses of NAP/ESO in JIA patients aged 12 to 16 years. Forty-six children and adolescents with JIA by International League of Associations for Rheumatology criteria, mean age of 13.6 years, from 18 US sites were prospectively enrolled over 2 years and followed for up to 6 months. Doses of the NAP/ESO fixed combination were based on baseline weight. The exploratory efficacy outcome was assessed with the ACR Pediatric-30, − 50, − 70, − 90 Response and the Childhood Health Assessment Questionnaire (CHAQ) discomfort and functional scores at months 1, 3, and 6 as change from baseline. Occurrence and causality were assessed for treatment emergent AEs (TEAEs) and discontinuations were monitored monthly.

**Results:**

Forty-six patients received at least 1 dose of naproxen/esomeprazole and 36 completed the trial. Thirty-seven (80.4%) had at least 1 treatment emergent adverse event (TEAE) and, with the exception of 2 events in one patient, all of the TEAEs were mild or moderate. Frequent TEAEs (≥5% of patients) were upper respiratory tract and gastrointestinal related. Eleven (23.9%) had at least 1 TEAE considered to be related to study drug. Four patients (8.7%) discontinued due to a TEAE with one of these being the only serious AE reported, acute hepatitis. Mean number of active joints at baseline was 3.1. Improvement in JIA signs and symptoms occurred at most assessments and by month 6, the percentage of patients with an ACR Pediatric-30, − 50, − 70, and − 90 Response was 47.1, 38.2, 32.4, and 17.6%, respectively. The percent of patients achieving ACR Pediatric response increased over time. CHAQ discomfort improved at each assessment and functional scores improved at all assessments for ‘Arising, Walking, and Activities’ with several improved for ‘Dressing and Grooming, Eating, Hygiene, and Grip’. There was no indication of a dose-related efficacy effect.

**Conclusion:**

NAP/ESO was well tolerated in JIA patients aged 12 to 16 years with high levels of response to ACR criteria. No new safety signals were identified for the well-characterized components of this fixed dosed JIA treatment, which was developed to reduce the risk of gastric ulcers.

**Trial registration:**

Clinicaltrials.gov, NCT01544114. Registered February 21, 2012.

**Electronic supplementary material:**

The online version of this article (10.1186/s12969-018-0260-y) contains supplementary material, which is available to authorized users.

## Background

Juvenile idiopathic arthritis (JIA) is an inflammatory arthritis of unknown etiology, which lasts for greater than 6 weeks with onset before 16 years of age [1, 2]. Per the American College of Rheumatology (ACR) guidelines, JIA is the most common chronic rheumatic disease in children [3]. Non-steroidal anti-inflammatory drugs (NSAIDs) have been the mainstay of initial management with naproxen being frequently prescribed, but they can cause serious side effects such as gastric ulcers. The ACR recommends NSAIDs and/or intra-articular steroids as first line therapy for oligoarticular JIA and as supplemental to other primary therapies [3, 4]. Monotherapy has been suggested to be successful in children less than 8 years of age, with low levels of disease burden [5]. However, children of all ages generally tolerate NSAIDs better than adults, but naproxen’s use in JIA is associated with gastrointestinal (GI) events in over 36% of patients, which can be a limiting factor for its use [6, 7]. Naproxen is approved by the Food and Drug Administration (FDA) for pediatric patients ≥2 years of age with JIA in dosages not greater than 15 mg/kg/day.

For pediatric patients up to 17 years of age, a delayed-release form of esomeprazole magnesium is approved in many countries for the short-term treatment (8 weeks) of gastroesophageal reflux disease and has been shown to be safe and effective [8]. An immediate-release (IR) form of esomeprazole magnesium was developed to allow for sequential gastric release of esomeprazole just prior to that of naproxen to provide maximum gastroprotective effects. A fixed combination product of this IR esomeprazole magnesium and naproxen was then developed and approved for use in adults with rheumatoid arthritis, osteoarthritis, and ankylosing spondylitis, and in adolescents with JIA, to reduce the incidence of NSAID related gastric ulcers. Herein, we report the first results of a JIA clinical efficacy and safety trial of three different doses of a combination product of naproxen with 20 mg of IR esomeprazole.

## Methods

### Study design

**Fig. 1:**
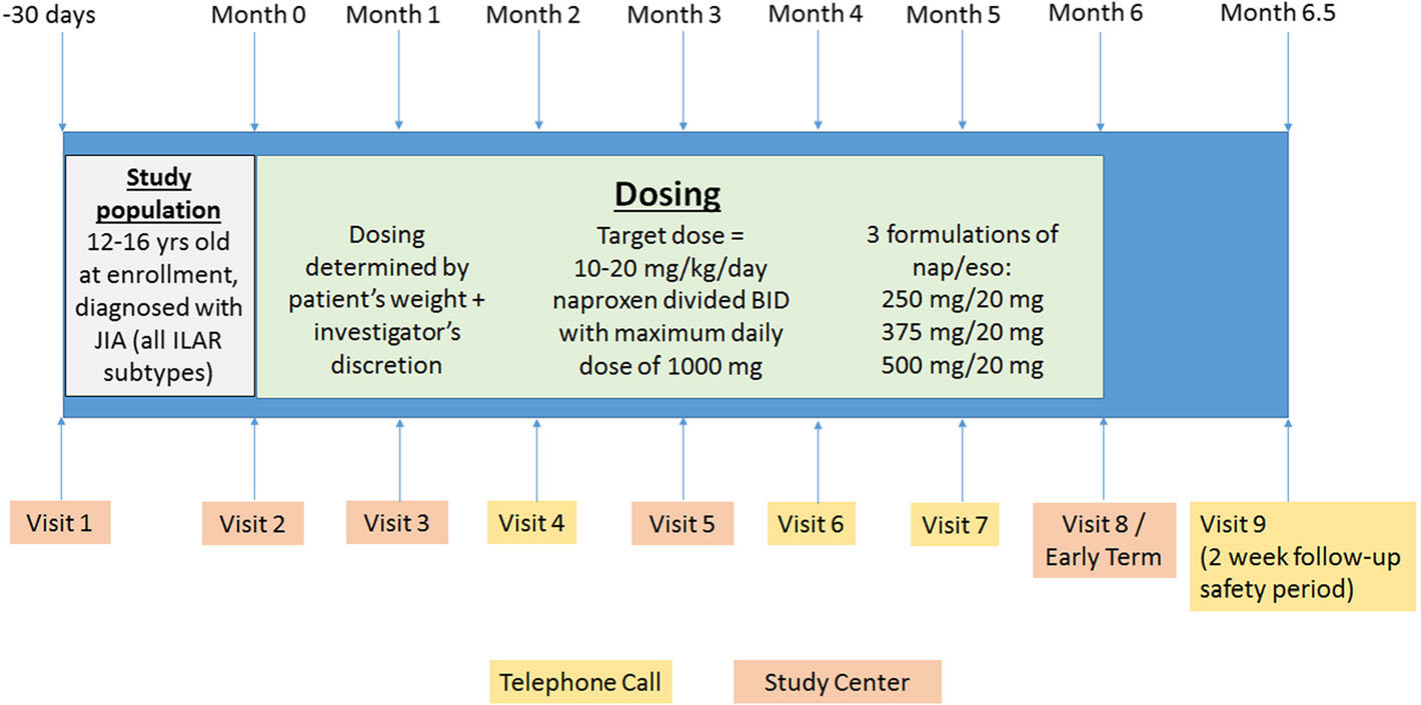
Overview of Study Design. Visits 1 and 2 could have been combined if results from all assessments at Visit 1 were obtained at the time of Visit 1. If Visits 1 and 2 were conducted on the same day, visit procedures that were specific to Visit 2 were also conducted during Visit 1. The telephone call during the 2-week safety follow-up period was required for all patients (i.e., patients who completed the full 6 months of treatment, patients who completed less than 6 months of treatment, patients who discontinued early from the study, and patients who took at least 1 dose of study drug)

The study was a phase 4, US only, multicenter, open-label, single arm, non-comparator study (Fig. 1) designed to evaluate the safety of 250 mg, 375 mg, or 500 mg of naproxen combined with 20 mg of esomeprazole in a fixed combination (NAP/ESO) given twice a day (BID) 30 min prior to the morning and evening meals for up to 6 months.

Adolescent male and female patients, age 12 to 16 years (inclusive), with an established diagnosis of JIA as defined by the International League of Associations for Rheumatology criteria were eligible for enrollment [1]. Patients who turned 17 years of age during the study were allowed to continue in the study. Enrollment of a sufficient number of patients to obtain 45 evaluable patients at approximately 20 study sites was planned, with evaluable patients defined as those who took at least 1 dose of study drug.

The Screening period was up to 30 days, and the Treatment period with open-label NAP/ESO was up to 6 months with a 2-week follow-up period for assessment of safety, for a total of ‘in study’ time of 7.5 months. Patients were screened for eligibility during Visit 1 (Screening visit) after the signed informed consent and assent were obtained. Medical history and prior/concomitant medication history were obtained, a complete physical examination (blood pressure [BP], pulse rate, oral temperature, height and weight), 12-lead electrocardiogram (ECG), eye exams, and blood and urine samples for determination of clinical chemistry (including serum iron/total iron binding capacity, vitamin B12 and magnesium), hematology and urinalysis were collected at Screening.

Screening (Visit 1) and entry into the Treatment period (Visit 2, Day 1) could have occurred during the same visit if the site performed all study Visit 1 and Visit 2 procedures within the same day, and if laboratory results were available to assess a patient’s eligibility during that visit. Patients were dispensed study drug only after all Visit 1 and Visit 2 procedures were performed and eligibility was confirmed. Patients returned to the study site for scheduled visits at the end of Months 1, 3, and 6 (±7 days), and were contacted via scheduled telephone calls at Months 2, 4, and 5 (±7 days). After the end of the Treatment period, safety was assessed by telephone after a 2-week follow-up period.

Safety assessments (adverse events [AEs], serious AEs [SAEs], concomitant medications, physical examinations with vital signs, weight, clinical chemistry and hematology) were performed at each scheduled visit. Ophthalmologic examinations were performed in accordance with the American Academy of Pediatrics guidelines [9]. AEs, SAEs, and concomitant medications were also assessed during telephone calls. Efficacy assessments were collected at baseline and at Months 1, 3, and 6. Blood samples for naproxen and esomeprazole PK analysis were collected for frequent sampling (Month 1, up to 8 patients) or sparse sampling (Months 1 and 3, remaining patients).

Study drug was dispensed at the start of the Treatment period and at Months 1 and 3, and unused study drug was collected at Months 1, 3, and 6. The NAP/ESO strength for each patient was determined by the patient’s weight at baseline (Table 1) and the Investigator’s discretion according to clinical guidelines on naproxen’s use [10]. The same dose assigned at baseline was maintained throughout the study. The naproxen and esomeprazole dosages are consistent with the previously identified safe and effective weight/age-appropriate doses administered to adolescent patients [8, 10, 11]. Study drug compliance was monitored at each visit by tablet counts from the returned bottles. Total tablets taken were defined as the difference of the returned from the total number dispensed. Any patient taking less than 80% or more than 120% of the assigned study drug was considered non-compliant.

**Table 1:** Minimum and Maximum Study Drug Dose (naproxen/esomeprazole magnesium) by Weight Group

Weight at Enrollment (kg)^a^	Minimum Dose^b^	Maximum Dose^b^
< 38	250 mg / 20 mg	250 mg / 20 mg
38 - < 50	250 mg / 20 mg	375 mg / 20 mg
50 - < 75	375 mg / 20 mg	500 mg / 20 mg
≥75	500 mg / 20 mg	500 mg / 20 mg

^a^Based on typial day-to-day fluctuations in body weight, a ± 3% window for body weight was permitted and used at the discretion of the Investigator when assigning the initial dose group

^b^Study drug (naproxen/esomeprazole magnesium) dose, twice daily (BID)

### Main inclusion criteria (full criteria provided in supplement)

1. Patients were male or female adolescents age 12 to 16 years at the time of enrollment.
2. Patient was diagnosed with JIA, including all the ILAR JIA subtypes: oligoarthritis, polyarthritis (both RF+ and RF-), psoriatic arthritis, enthesitis-related arthritis, undifferentiated arthritis, and systemic arthritis (those with presence of fever, rheumatoid rash, serositis, lymphadenopathy, macrophage activation syndrome in the past 6 months were excluded).
3. Patients who do not require ad hoc use of either active ingredient (naproxen or esomeprazole)
4. Patient’s body weight was > 31 kg and within the 5th to 95th percentile of body mass index (BMI) for age.

### Main exclusion criteria (full criteria listed in supplement)

1. Received treatment with any investigational agent 12 weeks or 5 half-lives of the investigational drug (whichever was longer) prior to Visit 2.
2. Had systemic JIA with presence of fever, rheumatoid rash, serositis, lymphadenopathy, macrophage activation syndrome within 6 months prior to start of study treatment.
3. Were receiving current treatment (i.e., within 4 weeks prior to start of study treatment) with naproxen > 20 mg/kg/day or > 1000 mg total daily dose.
4. Had any significant unstable hepatic, renal, pulmonary, ophthalmologic, neurologic or any other medical conditions indicated by medical/surgical history, physical or laboratory examination which would have confounded the study or put the patient at risk.
5. Had uncontrolled hypertension [12].

### Prior and Concomitant Therapy

Use of the following drugs was allowed during the study:

- Concomitant JIA medications, including anti-tumor necrosis factors, as long as the dosing regimen was stable for 1 month prior to enrollment.
- Acetaminophen and glucocorticoid intra-articular injections on an as needed basis.
- Corticosteroids, limited to ≤10 mg or 0.2 mg/kg prednisone equivalent per day (whichever was less).
- Methotrexate treatment ≤25 mg/week or 15 mg/m^2^/week (whichever was less).

Use of the following drugs was prohibited during the study:

- Treatment with another NSAID including additional naproxen at enrollment and during the study. NSAIDs, other than the study drug were prohibited during the study.
- Continuous treatment with antacids, H2-receptor antagonists, or proton pump inhibitor (PPI), in addition to the NAP/ESO received during this study.
- Continuous treatment with antifungals, antiretroviral drugs (atazanavir, nelfinavir, saquinavir), cilostazol, or warfarin (Coumadin®) or the use of these agents at any time between Visit 1 (Screening visit) and the final study visit (Visit 8 or ET visit). Concomitant use of the listed drugs is not recommended with esomeprazole.

Other medications, including over-the-counter medications and non-prescription dietary supplements that were considered necessary for the patient’s safety and well-being, were permitted at the discretion of the Investigator. Patients who were on prohibited medications could participate in the study if they discontinued their medication within 24 h of Visit 2, if deemed medically appropriate. In addition to the prohibited/restricted medications, addition of new treatment, treatment discontinuation, and change of dosage/administration was to be avoided as much as possible.

### Primary study endpoints

The primary objective was to evaluate the safety and tolerability of NAP/ESO in adolescents aged 12 to 16 years inclusive, with JIA, including the incidence and severity of adverse events (AEs) and serious AEs (SAEs), a change from baseline in vital signs, physical examination results and clinical laboratory results as well as serum iron/TIBC, vitamin B12 and magnesium levels.

Drug discontinuations were monitored monthly.

### Secondary objective

The pharmacokinetic (PK) characteristics of NAP/ESO in adolescents aged 12 to 16 years, inclusive, with JIA were evaluated and compared to historically reported adult and pediatric PK data of the individual components. Overall exposure was calculated for esomeprazole and exposure to naproxen was described by an analysis of trough levels, since naproxen is slowly absorbed and excreted over time. PK parameters were compared with that published previously for adult and pediatric patients.

### Exploratory efficacy objective

Disease activity was assessed with the ACR Pediatric response scores derived from the following:

- Physician’s global assessment of disease activity during the last 24 h prior to a visit was recorded using a 21-numbered circle visual analog scale (VAS) in 0.5 increments anchored by 0 = “no activity” and 10 = “maximum activity.” The change from baseline and percentage change from baseline was calculated at each scheduled post-baseline visit.
- CHAQ global assessment of well-being was rated for each patient by their parent at each scheduled visit using a 21-numbered VAS in 0.5 unit increments anchored by 0 = “very well” and 10 = “very poor” [13].
- CHAQ disability index score was calculated at each scheduled visit based on a 4-point Likert scale of 0 = “without any difficulty,” 1 = “with some difficulty,” 2 = “with much difficulty,” and 3 = “unable to do” as the mean of the non-missing functional area scores. If more than 2 of the 8 functional area scores were missing, then the disability index score was considered missing (i.e., a minimum of 6 functional area scores had to be non-missing).
- Number of joints with active arthritis was recorded. A joint was considered to have active arthritis if the patient had any of the following for the joint:
- Swelling, and/or
- Loss of motion and pain on motion, and/or
- Loss of motion and tenderness

Right and left joints were considered separately (e.g., both right and left wrists could have had active arthritis and would be counted as 2 joints with active arthritis).

- Number of joints with limited range of motion was recorded. A joint was considered to have limited range of motion if the patient has any of the following for that joint:
- Loss of motion and pain on motion, and/or
- Loss of motion and tenderness.

Right and left joints were considered separately (e.g., both right and left wrists could have had limited range of motion and would be counted as 2 joints with limited range of motion).

-Either serum C-reactive protein (CRP) or erythrocyte sedimentation rate (ESR). The test done at baseline was to have been continued at post-baseline visits, to assess change from baseline.

CHAQ discomfort index was also rated for each patient by their parent at each scheduled visit using a 21-numbered VAS in 0.5 unit increments anchored by 0 = “no pain” to 10 = “very severe pain.”

CHAQ Functional area scores. Eight functional areas were assessed (dressing and grooming, arising, eating, walking, hygiene, reach, grip, and activities) on a Likert scale of 1 to 4. For analysis purposes, responses were recoded using a scoring system of 0 = “without any difficulty,” 1 = “with some difficulty,” 2 = “with much difficulty” and 3 = “unable to do” to be consistent with the CHAQ scoring defined by Singh et al. [14].

### Analyses

As this was an open-label study, no hypothesis testing was performed. All safety and efficacy endpoints were summarized by descriptive statistics. For categorical variables, counts and percentages were presented. For continuous variables, the following were presented: n, mean, median, standard deviation, minimum, and maximum. Change from baseline values were calculated as the visit value minus the baseline value. Negative mean numbers of the individual changes indicated an improvement from baseline.

For the PK analysis, summary statistics of trough naproxen plasma concentration (lowest plasma concentration from pre-dose to 3 h post-dose) during steady state were presented by dose group and visit. Empirical Bayes’ estimates of the individual PK parameters were generated based on the final structural and variance parameter estimates and the individual plasma concentration measurements using nonlinear mixed effects modeling (NONMEM). Esomeprazole PK parameter estimates derived from this Population (Pop) PK analysis were analyzed and listed by study dose group and individual patient. The geometric %CV was calculated as 100 × √[exp(s^2^)-1], where s was the standard deviation of the data on a natural log scale.

## Results

### Disposition and demographics

Fifty-one patients were enrolled at 18 US sites over 2 years. Forty-six patients were assigned treatment. Forty-four patients received at least 1 dose of study drug and had at least 1 post-baseline assessment of any of the study parameters. Thirty-six (78.3%) of the treated patients received 6 months of study drug (defined as ≥166 days on study drug). Mean drug exposure was 5.5 months. See patient disposition in Table 2. Mean age, weight, and number of active joints at baseline were 13.6 years, 55.2 kg and 3.1, respectively (Table 3).

**Table 2:** Patient Disposition

	Number (%) of Patients			
	Naproxen/Esomeprazole Magnesium 250 mg/20 mg	Naproxen/Esomeprazole Magnesium 375 mg/20 mg	Naproxen/Esomeprazole Magnesium 500 mg/20 mg	Total
Enrolled				51
Not assigned treatment (eligibility criteria not fulfilled)				5
Assigned treatment	4	20	22	46
-Received study drug	4 (100)	20 (100)	22 (100)	46 (100)
-Completed study and received 6 mo of study drug	3 (75.0)	16 (80.0)	17 (77.3)	36 (78.3)
-Discontinued prematurely	1 (25.0)	4 (20.0)	5 (22.7)	10 (21.7)
-Adverse event	1 (25.0)	1 (5.0)	2 (9.1)	4 (8.7)
-Lost to follow-up	0	2 (10.0)	0	2 (4.3)
-Severe non-compliance with protocol	0	1 (5.0)	0	1 (2.2)
-Withdrew consent	0	0	3 (13.6)	3 (6.5)

**Table 3:** Demographics and Baseline Characteristics

	Naproxen/Esomeprazole Magnesium 250 mg/20 mg (*N* = 4)	Naproxen/Esomeprazole Magnesium 375 mg/20 mg (*N* = 20)	Naproxen/Esomeprazole Magnesium 500 mg/20 mg (*N* = 22)	Total (*N* = 46)
Age (years)				
Mean	12.8	13.6	13.8	13.6
SD	0.96	1.47	1.33	1.37
Median	12.5	13.0	13.5	13.0
Min, Max	12, 14	12, 16	12, 16	12, 16
Sex, n (%)				
Female	3 (75.0)	15 (75.0)	15 (68.2)	33 (71.7)
Male	1 (25.0)	5 (25.0)	7 (31.8)	13 (28.3)
Race, n (%)				
White	4 (100)	15 (75.0)	17 (77.3)	36 (78.3)
Black or African American	0	3 (15.0)	2 (9.1)	5 (10.9)
Asian	0	0	2 (9.1)	2 (4.3)
Other	0	2 (10.0)	1 (4.5)	3 (6.5)
Weight (kg)				
Mean	39.3	53.8	59.4	55.2
SD	2.63	9.37	8.27	10.07
Median	38.5	50.5	58.0	54.5
Min, Max	37, 43	42, 76	49, 76	37, 76
Height (cm)				
Mean	157.5	158.6	162.5	160.4
SD	4.73	8.78	7.70	8.14
Median	156.0	156.5	159.5	158.5
Min, Max	154, 164	144, 175	152, 176	144, 176
BMI (kg/m^2^)				
Mean	15.86	21.34	22.56	21.45
SD	1.579	2.797	3.476	3.534
Median	15.41	20.42	21.69	20.75
Min, Max	14.5, 18.1	16.8, 26.6	17.3, 32.0	14.5, 32.0
No. of Joints With Active Arthritis^a^				
N	4	19	21	44
Mean	1.0	4.1	2.5	3.1
SD	1.15	10.56	3.84	7.39
Median	1.0	1.0	1.0	1.0
Min, Max	0, 2	0, 46	0, 16	0, 46

*BMI* body mass index, *max* maximum, *min* minimum, *SD* standard deviation

^a^A joint was considered to have active arthritis if the patient had any of the following: swelling, loss of motion and pain on motion, and/or loss of motion and tenderness. Right and left joints were considered separately (e.g., both right and left wrists could have had active arthritis and would have been counted as 2 joints with active arthritis)

Five patients received glucocorticoids for JIA or “arthritis” related reasons during the study. Three patients received a total of 4 steroid joint injections. Two of the patients receiving injections also received low dose (≤ 10 mg) oral prednisone (one for only one day and the other on numerous days), 1 received oral prednisone from 5 to 80 mg on multiple days, and 1 received 7.5 mg oral prednisone on one day. Of the concomitant disease modifying agents of interest, 26 (56.5%) patients were on 1 or more of the following immunomodulators: methotrexate, adalimumab, infliximab, tocilizumab, abatacept, etanercept, leflunomide, rituximab, sulfasalazine, hydroxychloroquine sulfate. During the study, 11 patients either started new, switched immunomodulators or had their dose changed.

### Primary outcomes

- Thirty-seven patients (80.4%) had at least 1 TEAE (Table 4), as defined by the Medical Dictionary for Regulatory Activities (MedDRA). Frequent TEAEs (≥5%) were upper respiratory tract infection, upper abdominal pain, sinusitis, diarrhea, headache, nausea, and ligament sprain. Four patients (8.7%) discontinued due to a TEAE (1 SAE of hepatitis and 3 non-serious events; 1 patient with numbness, 1 patient with abdominal pain/dyspepsia, and 1 patient with worsening of JIA).
- All of the TEAEs were mild or moderate, with the exception of a 13 year old female, who had a SAE of acute hepatitis and a severe non-serious event of abnormal liver function tests. The patient had a history of hepatic steatosis (by liver biopsy), abdominal discomfort, parotitis, and jaw pain. Notably, the patient was not on concomitant methotrexate or other immunomodulators. On Day 22 of study drug treatment, the patient experienced dramatically increased AST and ALT compared to baseline. On Day 85, serologic testing was non-reactive for hepatitis A, B, and C. Study drug was permanently discontinued on Day 88 and the patient was withdrawn from the study. After withdrawal, the patient was hospitalized for a liver biopsy, which showed mixed inflammatory changes involving both hepatocytes and bile ducts in portal triads. A diagnosis of acute hepatitis with a possible etiology of drug induced or biliary cirrhosis was given. Patient was further treated in hospital and recovered. Both events were considered resolved on Day 157, with the hepatitis considered reasonably related to the study drug. Gastrointestinal AEs were reported in 17 patients, most frequently upper abdominal pain, diarrhea and nausea. (Table 4). There were no severe or serious GI events. Of these 17 patients, 7 were on concomitant disease modifying agents (methotrexate, infliximab, leflunomide and tocilizumab).
- A total of 11 (23.9%) patients had at least 1 TEAE considered by the Investigator to be related to the study drug and 3 discontinued due to TEAE considered to be related to the study drug (hepatitis, numbness, and abdominal pain/dyspepsia).
- The only study drug-related TEAE that occurred in ≥5% of all patients was upper abdominal pain (3 [6.5%] patients).
- Due to a theoretical risk of decreased levels for iron, vitamin B12, and magnesium during long-term PPI exposure, potential alterations in these moieties during 6 months of PPI therapy were evaluated and no significant changes were seen.

**Table 4:** TEAEs occurring in at least 2 patients

	Number (%) of Patients			
MedDRA System Organ Class Preferred Term	NAP/ESO 250 mg/20 mg (*N* = 4)	NAP/ESO 375 mg/20 mg (*N* = 20)	NAP/ESO 500 mg/20 mg (*N* = 22)	Total (*N* = 46)
Patient with any TEAE	4 (100)	16 (80.0)	17 (77.3)	37 (80.4)
Gastrointestinal disorders	1 (25.0)	8 (40.0)	8 (36.4)	17 (37.0)
Abdominal pain upper	0	3 (15.0)	2 (9.1)	5 (10.9)
Diarrhea	0	2 (10.0	2 (9.1)	4 (8.7)
Nausea	0	3 (15.0)	1 (4.5)	4 (8.7)
Abdominal discomfort	0	2 (10.0)	0	2 (4.3)
Dyspepsia	0	0	2 (9.1)	2 (4.3)
Vomiting	0	1 (5.0)	1 (4.5)	2 (4.3)
Infections and infestations	2 (50.0)	4 (20.0)	9 (40.9)	15 (32.6)
Upper respiratory tract infection	1 (25.0)	2 (10.0)	6 (27.3)	9 (19.6)
Sinusitis	0	1 (5.0)	4 (18.2)	5 (10.9)
Gastroenteritis viral	0	0	2 (9.1)	2 (4.3)
Tooth infection	0	1 (5.0)	1 (4.5)	2 (4.3)
Musculoskeletal and connective tissue disorders	2 (50.0)	2 (10.0)	2 (9.1)	6 (13.0)
Back pain	1 (25.0)	1 (5.0)	0	2 (4.3)
Pain in extremity	0	1 (5.0)	1 (4.5)	2 (4.3)
Injury, poisoning and procedural complications	0	2 (10.0)	3 (13.6)	5 (10.9)
Ligament sprain	0	0	3 (13.6)	3 (6.5)
Nervous system disorders	2 (50.0)	1 (5.0)	2 (9.1)	5 (10.9)
Headache	1 (25.0)	1 (5.0)	2 (9.1)	4 (8.7)
General disorders and administration site conditions	0	0	2 (9.1)	2 (4.3)
Fatigue	0	0	2 (9.1)	2 (4.3)
Immune system disorders	0	1 (5.0)	1 (4.5)	2 (4.3)
Hypersensitivity	0	1 (5.0)	1 (4.5)	2 (4.3)
Neoplasms benign, malignant and unspecified (incl. cysts and polyps)	1 (25.0)	1 (5.0)	0	2 (4.3)
Skin papilloma	1 (25.0)	1 (5.0)	0	2 (4.3)
Respiratory, thoracic and mediastinal disorders	0	1 (5.0)	1 (4.5)	2 (4.3)
Cough	0	1 (5.0)	1 (4.5)	2 (4.3)

### Secondary outcomes- pharmacokinetics

Forty patients supplied samples for esomeprazole and 41 provided samples for naproxen. Esomeprazole concentrations observed in adolescents are comparable to those previously reported in adult and adolescent studies [15]. Geometric mean values of esomeprazole CL/F and V/F were higher in this pediatric study compared to the estimates in adult (CL/F: 24.66 vs. 12.79 L/h/70kg^0.75^; V/F: 38.68 vs. 21.03 L/70 kg). Individual oral CL/F estimates in adolescent subjects (ranging from 3.84 to 131.91 L/h/70kg^0.75^) were within the range of estimates observed in adults (3.43–136.62 L/h/70kg^0.75^) [15]. Total esomeprazole exposures (estimated as AUCs) ranged from 0.51 to 19.69 μmol·h/L in adolescent subjects and from 0.46 to 14.68 μmol·h/L in adult healthy volunteers, and are comparable to previously reported values in the literature [8, 15–17]. Naproxen C_max_ and T_max_ data in frequently sampled pediatric patients were comparable to those reported in pediatric studies [18, 19] and adult studies [20]. Naproxen concentrations in terms of range and variability in this study were comparable to what was previously reported in children [18]. See supplemental materials for summary of naproxen and esomeprazole plasma concentration frequent and sparse sampling groups (Additional file 1: Tables S1 and S2).

### Efficacy assessment

**Fig. 2:**
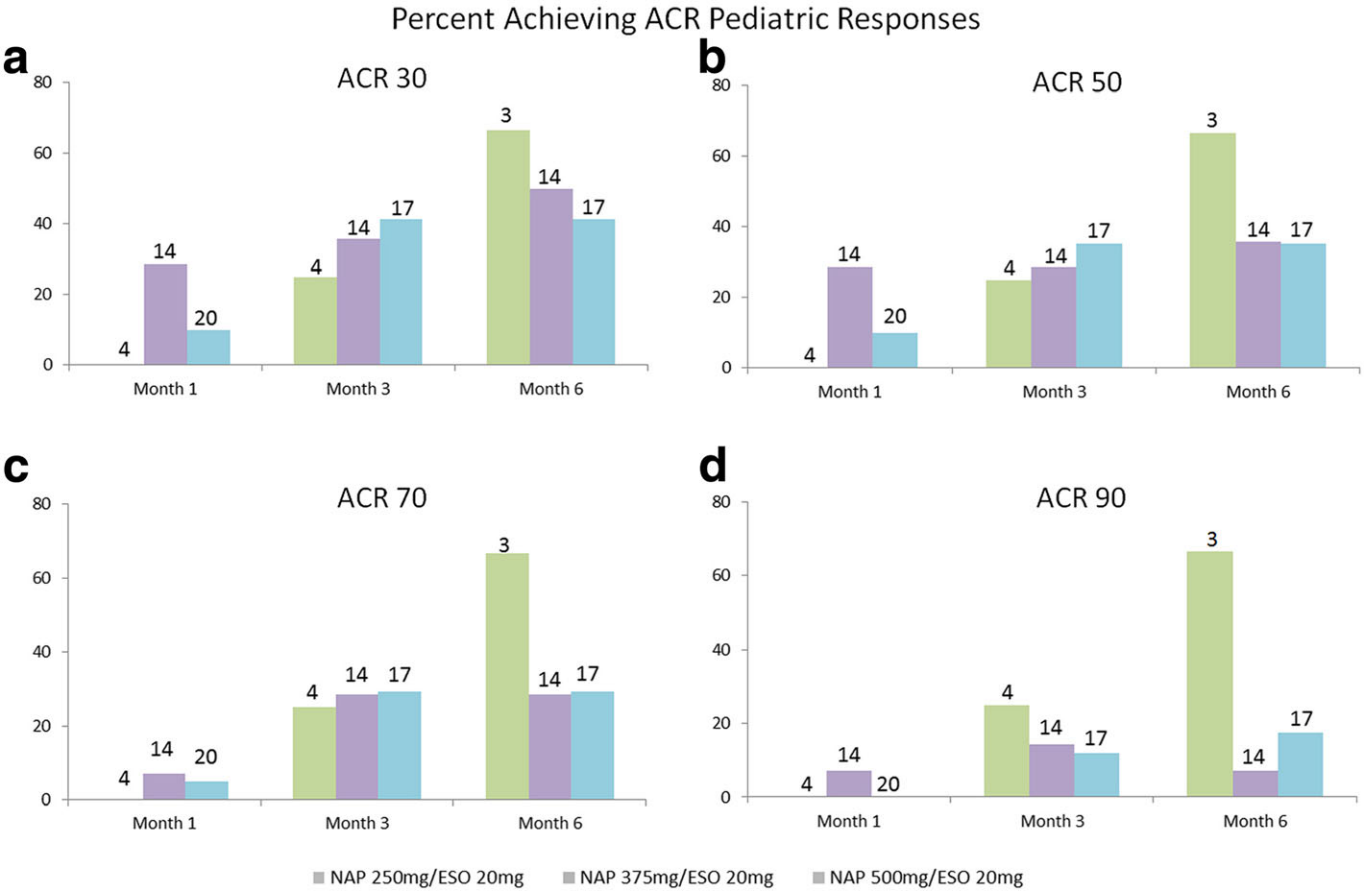
ACR Scores. The number above each bar represents the number of patients at that dose. The four patients in the lowest dose group did not reach any ACR response at Month 1. The ACR Pediatric-30, −50, −70, and − 90 responses were defined as an improvement of at least 30% (or 50, 70, 90%, respectively) from baseline in at least 3 of the 6 signs and symptoms variables, with no more than 1 of the remaining variables worsening by > 30%. JIA signs and symptoms variables: physician’s global assessment of disease activity, CHAQ disability index score, CHAQ global assessment of well-being, number of joints with active arthritis, number of joints with limited range of motion, serum CRP or ESR

- The percentage of patients achieving ACR Pediatric response increased over time (Fig. 2).
- The mean baseline score for physician’s assessment of disease activity was 2.58 and individual mean improvement from baseline was − 0.67, − 0.99, and − 1.23 at Months 1, 3, and 6, respectively.
- Mean CHAQ global assessment of Well-Being baseline score was 3.43 and mean improvement from baseline was − 0.80, − 0.93, and − 1.39 at Months 1, 3, and 6.
- Mean CHAQ functional scores indicated improvement from baseline for 3 of the functional areas (arising, walking, and activities). For 4 of the functional areas (dressing and grooming, eating, hygiene, and grip), the mean change from baseline indicated an improvement or no change (mean change 0.0) at each assessment. For the remaining functional area of ‘reach’, there was no change at each post-baseline assessment except Month 1 (mean change 0.2).
- The mean baseline score for the CHAQ disability index was 0.506. The mean improvement from baseline was − 0.069, − 0.064, and − 0.155 at Months 1, 3, and 6, respectively.
- The mean baseline number of joints with active arthritis was 3.1. The individual mean change indicated an improvement from baseline to each post-baseline assessment. The mean improvement from baseline was − 1.1, − 0.4, and − 0.6 at Months 1, 3, and 6, respectively.
- The mean baseline number of joints with limited range of motion was 1.7. The individual mean improvement from baseline was − 0.9, − 0.1, and − 0.2 at Months 1, 3, and 6, respectively.
- The mean baseline serum CRP was 8.807 mg/mL with a mean change of 0.169, − 1.076, and − 1.120 at Months 1, 3, and 6, respectively, indicating individual mean improvement at Months 3 and 6.
- The mean baseline serum ESR was 10.4 mm/hr. The mean individual change indicated improvement from baseline to Month 3. The mean change from baseline was 0.4, − 0.1, and 0.8 at Months 1, 3, and 6, respectively.
- CHAQ discomfort improved at each assessment from a baseline mean of 4.41. The mean improvement from baseline was − 1.24, − 1.21, and − 2.20 at Months 1, 3, and 6.
- There was no indication of a dose-related efficacy effect in any of the outcomes.
- Adherence with study medication was good with 73.9% of patients (34 of 46) with ≥80% and ≤ 120% compliance and 21.7% (10 of 46 patients) demonstrating < 80% or > 120% (non-compliance). Two subjects were lost to follow-up and therefore did not have an overall compliance calculated.

### Additional analyses

**Fig. 3:**
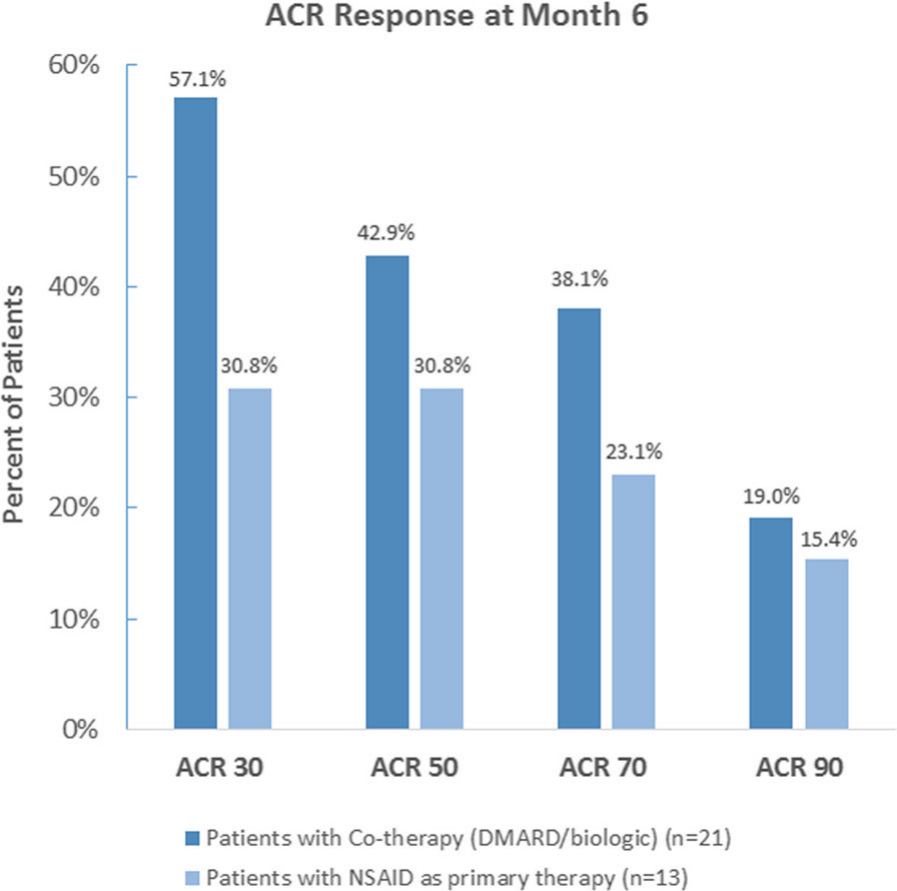
ACR Response in patients with co-therapy and in patients with NSAID as primary therapy at month 6

Twenty-six of the 46 patients who were assigned treatment with NAP/ESO were also taking a DMARD/biologic during the study and the remaining patients utilized NAP/ESO as their primary therapy. Those who were on co-therapy and had variables to determine a month 6 ACR response (*N* = 21) displayed a greater increase in percentage of patients with ACR Response 30, − 50, − 70, and − 90 when compared to those using NAP/ESO as their primary therapy (Fig. 3). Baseline demographics of the co-therapy group appeared similar to the primary therapy group (see Additional file 1: Table S3), and baseline disease measures showed a trend of increased severity in the co-therapy group (see Additional file 1: Table S4).

## Discussion

The fixed combination of naproxen and esomeprazole was well tolerated in this population of 12 to 16-year-old patients with JIA, and no new safety signals were identified. The safety results were consistent with the well-characterized profiles of the components; naproxen and esomeprazole magnesium.

Esomeprazole was selected as the PPI of choice for this combination because of its well-documented acid-inhibiting properties [21] and its proven efficacy in reducing the risk of NSAID-associated gastric ulcers [22]. Naproxen was chosen because historically, up to 85% of JIA patients have been treated with NSAIDs [23], with naproxen noted to be the predominate NSAID in use recently [5]. In a previous trial, naproxen was noted to produce GI AEs in 39.6% of patients with the event rate driven predominantly by abdominal pain in an adolescent patient population aged 12–17, similar to this study. The abdominal pain rate found in this study was 10.9% [24]. Research should continue on identifying which JIA patients will benefit the most from NSAID therapy as well as those most at risk for NSAID induced GI events who would benefit from gastroprotection with this fixed combination of naproxen and esomeprazole.

Our study found good results with naproxen/esomeprazole monotherapy with ACR 30 and 50 responses in 31% of our JIA patients. However, our data seem to indicate that patients with higher disease activity respond better to combination immunomodulator therapy and naproxen/esomeprazole, as evidenced by higher ACR 30 and 50 responses, though none of the baseline differences were statistically significantly different between the two groups.

At baseline, patients in this study were on mean approximately 14 years of age, had 3 active joints, and a CRP of about 9. Our patients demonstrated good responses over the observation period with 47% achieving an ACR-30, 38% receiving an ACR-50, 32% receiving an ACR-70 response, and 18% receiving an ACR-90 response at month 6. Improvements were observed in JIA signs and symptoms, the CHAQ discomfort index, and several CHAQ functional area scores. The percentage of patients that achieved an ACR Pediatric response increased over time during the study and were generally similar to historic NSAID study results indicating significant responses between 25 to 33% of patients [25].

There was no association of the weight based doses with response indicating that the doses chosen in this study were appropriate and in line with published guidelines [10]. We did not, however, titrate doses to determine optimal efficacy, but the doses used in this study (mean of ≈ 15.5 mg/kg/day) represent previously identified effective doses of naproxen for JIA treatment, with a target range of between 10 and 20 mg/kg/day divided twice daily with a maximum of 1000 mg/day. Only 4 patients received the lowest dose (250 mg NAP/20 mg ESO), and therefore there was limited data within the lowest weight group, leading to the currently recommended FDA approval of the fixed dosage form in those patients 12 years of age and older weighing at least 38 kg, requiring naproxen for symptomatic relief of arthritis and esomeprazole magnesium to decrease the risk of developing naproxen-associated gastric ulcers [26].

Mean drug exposure was approximately 165 days in the 46 patients, with 36 (78.3%) of them receiving 6 months of study drug (defined as ≥166 days on study drug). The PK results approximated the esomeprazole PK data from previously published pediatric studies. The naproxen C_max_ and T_max_ values in the more frequently sampled pediatric patients were comparable to those reported in the literature [18–20]. In addition, naproxen concentrations in terms of range and variability in this pediatric study were comparable to that previously reported in children. These results indicated that combining esomeprazole and naproxen did not result in any significant interaction affecting overall pharmacokinetics of the individual components of the fixed dosage form.

## Conclusions

The fixed combination of NAP/ESO was well tolerated in JIA patients aged 12 to 16 years. No new safety signals were identified for the well-characterized components of this fixed dosed JIA treatment, which was developed to reduce the risk of gastric ulcers in patients requiring chronic naproxen therapy. Improvement in JIA signs and symptoms occurred at most assessments and by month 6, the percentage of patients with an ACR Pediatric-30, − 50, − 70, and − 90 Response was 47.1, 38.2, 32.4, and 17.6%, respectively.

## Additional file

Additional file 1: Supplemental Data. (DOCX 54 kb)
