# Supplementary material for: A 6-month, multicenter, open-label study of fixed dose naproxen/esomeprazole in adolescent patients with juvenile idiopathic arthritis
Source: Pediatr Rheumatol Online J. 2018 Jun 26;16:41. doi: 10.1186/s12969-018-0260-y (PMC6019234; doi:10.1186/s12969-018-0260-y)
Supplement: Supplementary file 1 — Supplemental Data. (DOCX 54 kb) [file 12969_2018_260_MOESM1_ESM.docx]

**Additional file 1**

**Inclusion criteria**

1. Parent or legal guardian was able to provide written informed consent and patient was able to provide written assent if appropriate.

2. Patient was male or female adolescent age 12 to 16 years at the time of enrollment.

3. Patient was diagnosed with JIA, including all the ILAR JIA subtypes: oligoarthritis, polyarthritis (both RF+ and RF-), psoriatic arthritis, enthesitis-related arthritis, undifferentiated arthritis, and systemic arthritis (those with presence of fever, rheumatoid rash, serositis, lymphadenopathy, macrophage activation syndrome in the past 6 months were excluded).

4. Based upon Investigator judgment, it was determined appropriate for the patient to undergo up to 6 months of continuous treatment with NAP/ESO (delivering naproxen 10-20 mg/kg/day divided BID, with a maximum dose of 1000 mg/day). No as needed (PRN) usage of either excipient (naproxen/esomeprazole) was allowed.

5. Patient's body weight was >31 kg and within the 5th to 95th percentile of body mass index (BMI) for age.

6. Patient was able to swallow tablets whole with only liquid.

7. All female patients in a relationship capable of producing a pregnancy must have a negative urine pregnancy test and confirmed (by the Investigator) use of a highly effective form of birth control.

8. Patient clearly understood and was willing to comply with all study procedures and scheduled visits.

9. The Investigator believed participation in the study would not be harmful to the patient.

**Exclusion Criteria**

Patients were excluded if they:

1. Or their parents were involved in the planning and/or conduct of the study (applied to Sponsor staff or its representative and/or staff at the study site).

2. Were previously enrolled in the present study.

3. Received treatment with any investigational agent 12 weeks or 5 half-lives of the investigational drug (whichever was longer) prior to Visit 2.

4. Had systemic JIA with presence of fever, rheumatoid rash, serositis, lymphadenopathy, macrophage activation syndrome within 6 months prior to start of study treatment.

5. Were receiving current treatment (i.e., within 4 weeks prior to start of study treatment) with naproxen >20 mg/kg/day or >1000 mg total daily dose.

6. Had a hemoglobin ≤8.5 g/dL.

7. Had cardiovascular or cerebrovascular disease, based on history or risk factors, e.g., congestive heart failure, established ischemic heart disease, peripheral arterial disease, and/or cerebrovascular disease.

8. Had prior history of peptic ulcer complications, defined as lifetime history of peptic ulcer which had resulted in bleeding, obstruction, or perforation.

9. Had estimated baseline creatinine clearance of <60 mL/minute.

10. Had severe hepatic impairment (e.g., Child-Pugh C score).

11. Had known hypersensitivity to naproxen, esomeprazole, substituted benzimidazoles, or to any of the excipients.

12. Had aspirin-sensitive asthma.

13. Were receiving continuous treatment with systemic antifungals, antiretroviral drugs (such as atazanavir, nelfinavir, and saquinavir), cilostazol or warfarin (Coumadin®), or the use of these agents at any time between Visit 1 and the final evaluation visit.

14. Had any significant unstable hepatic, renal, pulmonary, ophthalmologic, neurologic, or any other medical conditions indicated by medical/surgical history, physical, or laboratory examination that might have confounded the study or put the patient at greater risk during study participation. This included any laboratory assessments (clinical chemistry, hematology, urinalysis) deemed clinically significant by the Investigator.

15. Had uncontrolled hypertension defined as systolic or diastolic BP that exceeded the 95th percentile for age and height [1].

16. Had coagulation disorders or were receiving drug therapy that interferes with hemostasis.

17. Were pregnant and/or lactating.

18. Had known history of human immunodeficiency virus, hepatitis B virus, or hepatitis C virus.

19. Had active uveitis. Patients with a history of uveitis, including those currently receiving stable treatment for at least 6 months and no active disease, were allowed. Ophthalmologic examinations were performed in accordance with the American Academy of Pediatrics guidelines [2].

**Table S1.** Summary of Naproxen Plasma Concentration (µg/mL), frequent and sparse sampling groups

| **Arm visit^1^**  **Statistic** | **Pre-dose** | **Time after dose intake (hours)^2^** | | | | | | **Trough^3^** |
| --- | --- | --- | --- | --- | --- | --- | --- | --- |
|  |  | **0.5** | **0.5 - 1** | **1** | **1.5** | **2 - 3** | **3** |  |
| **NAP/ESO 250mg/20mg** | | | | | | | | |
| **Month 1** | | | | | | | | |
| **N** | 1 | 1 | 2 | 1 | 1 | 1 | 1 | 4 |
| **Geometric mean** | 53.20 | 45.00 | 70.09 | 43.90 | 46.90 | 85.30 | 37.80 | 63.09 |
| **Geometric CV (%)** | - | - | 27.3 | - | - | - | - | 40.1 |
| **Arithmetic mean** | 53.20 | 45.00 | 71.35 | 43.90 | 46.90 | 85.30 | 37.80 | 66.45 |
| **SD** | - | - | 18.880 | - | - | - | - | 22.954 |
| **Median** | 53.20 | 45.00 | 71.35 | 43.90 | 46.90 | 85.30 | 37.80 | 71.35 |
| **Min, Max** | 53.2, 53.2 | 45.0, 45.0 | 58.0, 84.7 | 43.9, 43.9 | 46.9, 46.9 | 85.3, 85.3 | 37.8, 37.8 | 37.8, 85.3 |
| **Month 3** | | | | | | | | |
| **n** | - | - | - | - | - | 2 | - | 2 |
| **Geometric mean** | - | - | - | - | - | 63.16 | - | 63.16 |
| **Geometric CV (%)** | - | - | - | - | - | 67.3 | - | 67.3 |
| **Arithmetic mean** | - | - | - | - | - | 69.15 | - | 69.15 |
| **SD** | - | - | - | - | - | 39.810 | - | 39.810 |
| **Median** | - | - | - | - | - | 69.15 | - | 69.15 |
| **Min, Max** | - | - | - | - | - | 41.0, 97.3 | - | 41.0, 97.3 |
| **NAP/ESO 375 mg/20mg** | | | | | | | | |
| **Month 1** | | | | | | | | |
| **n** | 1 | 1 | 12 | 1 | 1 | 4 | 1 | 17 |
| **Geometric mean** | 83.40 | 74.30 | 18.69 | 67.30 | 71.70 | 16.23 | 56.30 | 19.29 |
| **Geometric CV (%)** | - | - | 1587.3 | - | - | 3239.4 | - | 1334.9 |
| **Arithmetic mean** | 83.40 | 74.30 | 51.94 | 67.30 | 71.70 | 46.65 | 56.30 | 50.95 |
| **SD** | - | - | 38.009 | - | - | 33.927 | - | 34.874 |
| **Median** | 83.40 | 74.30 | 57.10 | 67.30 | 71.70 | 55.65 | 56.30 | 56.30 |
| **Min, Max** | 83.4, 83.4 | 74.30, 74.30 | NA, 113.0 | 67.3, 67.3 | 71.7, 71.7 | 0.3, 75.0 | 56.3, 56.3 | NA, 113.0 |
| **Month 3** | | | | | | | | |
| **n** | - | - | 3 | - | - | 11 | - | 14 |
| **Geometric mean** | - | - | 30.12 | - | - | 35.03 | - | 33.91 |
| **Geometric CV (%)** | - | - | 147.8 | - | - | 687.2 | - | 476.0 |
| **Arithmetic mean** | - | - | 40.30 | - | - | 60.12 | - | 55.87 |
| **SD** | - | - | 27.455 | - | - | 28.713 | - | 28.659 |
| **Median** | - | - | 53.90 | - | - | 57.70 | - | 56.60 |
| **Min, Max** | - | - | 8.7, 58.3 | - | - | NA, 99.3 | - | NA, 99.3 |
| **NAP/ESO 500mg/20mg** | | | | | | | | |
| **Month 1** | | | | | | | | |
| **n** | 4 | 4 | 9 | 4 | 4 | 7 | 4 | 20 |
| **Geometric mean** | 10.15 | 9.27 | 71.18 | 9.32 | 32.68 | 47.95 | 50.93 | 40.71 |
| **Geometric CV (%)** | 12028.2 | 9904.4 | 35.5 | 9989.5 | 46.6 | 39.2 | 52.6 | 277.3 |
| **Arithmetic mean** | 37.23 | 32.68 | 75.08 | 32.85 | 34.98 | 50.81 | 55.60 | 57.51 |
| **SD** | 28.993 | 24.769 | 26.445 | 24.829 | 13.792 | 17.776 | 25.586 | 28.179 |
| **Median** | 39.55 | 35.60 | 65.50 | 35.85 | 35.45 | 49.70 | 55.25 | 56.80 |
| **Min, Max** | NA, 69.7 | NA, 59.4 | 42.4, 119.0 | NA, 59.6 | 17.9, 51.1 | 26.7, 70.3 | 29.1, 82.8 | NA, 119.0 |
| **Month 3** | | | | | | | | |
| **n** | - | - | 4 | - | - | 9 | - | 13 |
| **Geometric mean** | - | - | 17.25 | - | - | 59.58 | - | 40.69 |
| **Geometric CV (%)** | - | - | 2738.3 | - | - | 96.7 | - | 325.4 |
| **Arithmetic mean** | - | - | 49.19 | - | - | 77.13 | - | 68.54 |
| **SD** | - | - | 38.363 | - | - | 51.931 | - | 48.435 |
| **Median** | - | - | 57.50 | - | - | 85.70 | - | 78.20 |
| **Min, Max** | - | - | 0.4, 81.4 | - | - | 18.7, 150.0 | - | 0.4, 150.0 |

CV, coefficient of variation; Max, maximum; min, minimum; NA, not applicable; SD, standard deviation

NOTE: Where a result was less than the limit of quantification value (0.1 µg/mL) was used for the summary statistics. Where the minimum value was less than the limit of quantification, then the minimum value is reported as NA.

^1^Visit window has been applied for all unscheduled and early termination visits. ^2^Planned times of blood draw

^3^Trough concentration was defined as lowest plasma concentrations from pre-dose to 3 hours post-dose, for each individual patient

Table S2. Summary of Esomeprazole Plasma Concentrations (ng/mL), Frequent and Sparse Sampling Groups

| **Arm visit^1^**  **Statistic** | **Pre-dose** | **Time after dose intake (hours)^2^** | | | | | |
| --- | --- | --- | --- | --- | --- | --- | --- |
|  |  | **0.5** | **0.5 - 1** | **1** | **1.5** | **2 - 3** | **3** |
| **NAP/ESO 250mg/20mg** | | | | | | | |
| **Month 1** | | | | | | | |
| **n** | 1 | 1 | 2 | 1 | 1 | 1 | 1 |
| **Geometric mean** | 6.91 | 6.91 | 402.22 | 24.07 | 99.48 | 153.70 | 366.12 |
| **Geometric CV (%)** | - | - | 1327.8 | - | - | - | - |
| **Arithmetic mean** | 6.91 | 6.91 | 1045.35 | 24.07 | 99.48 | 153.70 | 366.12 |
| **SD** |  |  | 1364.539 |  |  |  |  |
| **Median** | 6.91 | 6.91 | 1045.35 | 24.07 | 99.48 | 153.70 | 366.12 |
| Min, Max | NA, 6.9 | NA, 6.9 | 80.5, 2010.2 | 24.1, 24.1 | 99.5, 99.5 | 153.7, 153.7 | 366.1, 366.1 |
| Month 3 | | | | | | | |
| **n** | - | - | - | - | - | 1 | - |
| **Geometric mean** | - | - | - | - | - | 10.09 | - |
| **Geometric CV (%)** | - | - | - | - | - | - | - |
| **Arithmetic mean** | - | - | - | - | - | 10.09 | - |
| **SD** | - | - | - | - | - | - | - |
| **Median** | - | - | - | - | - | 10.09 | - |
| **Min, Max** | - | - | - | - | - | 10.1, 10.1 | - |
| **NAP/ESO 375 mg/20mg** | | | | | | | |
| Month 1 | | | | | | | |
| **n** | 1 | 1 | 12 | 1 | 1 | 4 | 1 |
| **Geometric mean** | 6.91 | 31.85 | 67.56 | 209.31 | 262.85 | 25.08 | 121.93 |
| **Geometric CV (%)** | - | - | 298.4 | - | - | 742.5 | - |
| **Arithmetic mean** | 6.91 | 31.85 | 148.12 | 209.31 | 262.85 | 126.13 | 121.93 |
| **SD** | - | - | 176.664 | - | - | 231.432 | - |
| **Median** | 6.91 | 31.85 | 101.89 | 209.31 | 262.85 | 12.21 | 121.93 |
| **Min, Max** | NA, 6.9 | 31.8, 31.8 | NA, 639.0 | 209.3, 209.3 | 262.8, 262.8 | NA, 473.2 | 121.9, 121.9 |
| Month 3 | | | | | | | |
| **n** | - | - | 3 | - | - | 11 | - |
| **Geometric mean** | - | - | 59.41 | - | - | 54.96 | - |
| **Geometric CV (%)** | - | - | 1100.6 | - | - | 395.1 | - |
| **Arithmetic mean** | - | - | 204.82 | - | - | 167.64 | - |
| **SD** | - | - | 302.174 | - | - | 238.607 | - |
| **Median** | - | - | 54.92 | - | - | 60.10 | - |
| **Min, Max** | - | - | NA, 552.6 | - | - | NA, 683.9 | - |
| NAP/ESO 500mg/20mg | | | | | | | |
| Month 1 | | | | | | | |
| **n** | 4 | 4 | 9 | 4 | 4 | 7 | 4 |
| **Geometric mean** | 6.91 | 163.45 | 85.38 | 228.36 | 191.36 | 67.58 | 95.00 |
| **Geometric CV (%)** | 0.0 | 939.5 | 570.1 | 161.7 | 139.1 | 297.9 | 542.0 |
| **Arithmetic mean** | 6.91 | 360.94 | 309.29 | 333.05 | 271.74 | 160.29 | 200.33 |
| **SD** | 0.000 | 253.448 | 490.271 | 262.208 | 226.375 | 198.145 | 176.629 |
| **Median** | 6.91 | 433.48 | 56.30 | 343.15 | 242.30 | 44.56 | 207.24 |
| **Min, Max** | NA, 6.91 | NA, 569.9 | NA, 1499.0 | 51.8, 594.1 | 56.6, 545.7 | 13.2, 535.4 | NA, 379.9 |
| Month 3 | | | | | | | |
| **n** | - | - | 3 | - | - | 9 | - |
| **Geometric mean** | - | - | 258.64 | - | - | 26.19 | - |
| **Geometric CV (%)** | - | - | 318.2 | - | - | 199.4 | - |
| **Arithmetic mean** | - | - | 536.52 | - | - | 50.37 | - |
| **SD** | - | - | 697.052 | - | - | 56.438 | - |
| **Median** | - | - | 211.73 | - | - | 20.03 | - |
| **Min, Max** | - | - | 61.1, 1336.7 | - | - | NA, 170.6 | - |

CV, coefficient of variation; Max, maximum; min, minimum; NA, not applicable; SD, standard deviation

NOTE: Where a result was less than the limit of quantification value (0.1 µg/mL) was used for the summary statistics. Where the minimum value was less than the limit of quantification, then the minimum value is reported as NA.

^1^Visit window has been applied for all unscheduled and early termination visits

^2^Planned times of blood draw

**Table S3**. Baseline characteristics of patients on co-therapy vs those with NSAID as primary therapy

| **Baseline Demographic** | **All Patients** | **Patients on Co-Therapy** | **Patients with NSAID as Primary Therapy** |
| --- | --- | --- | --- |
| **Age (years)** |  |  |  |
| n | 46 | 26 | 20 |
| Mean | 13.6 | 13.6 | 13.6 |
| SD | 1.37 | 1.39 | 1.39 |
| Median | 13.0 | 13.5 | 13.0 |
| Min, Max | 12, 16 | 12, 16 | 12, 16 |
| **Weight (kg)** |  |  |  |
| n | 46 | 26 | 20 |
| Mean | 55.2 | 54.1 | 56.7 |
| SD | 10.07 | 9.56 | 10.77 |
| Median | 54.5 | 54.5 | 55.0 |
| Min, Max | 37, 76 | 37, 76 | 38, 76 |
| **Height (cm)** |  |  |  |
| n | 46 | 26 | 20 |
| Mean | 160.4 | 158.5 | 162.9 |
| SD | 8.14 | 7.00 | 9.00 |
| Median | 158.5 | 158.0 | 161.0 |
| Min, Max | 144, 176 | 145, 171 | 144, 176 |
| **BMI (kg/m^2)** |  |  |  |
| n | 46 | 26 | 20 |
| Mean | 21.45 | 21.58 | 21.27 |
| SD | 3.527 | 3.893 | 3.076 |
| Median | 20.75 | 20.50 | 21.50 |
| Min, Max | 14.5, 32.0 | 14.5, 32.0 | 15.2, 26.7 |
| **Assigned study drug dose (naproxen component, mg/kg/day)** |  |  |  |
| n | 46 | 26 | 20 |
| Mean | 15.53 | 15.62 | 15.41 |
| SD | 2.666 | 2.546 | 2.876 |
| Median | 15.96 | 15.59 | 15.44 |
| Min, Max | 9.87, 20.41 | 9.87, 19.61 | 11.03, 20.41 |

**Table S4**. Baseline JIA disease measures of patients on co-therapy vs those with NSAID as primary therapy

| **Baseline measure** | **All Patients** | **Patients on Co-Therapy** | **Patients with NSAID as Primary Therapy** |
| --- | --- | --- | --- |
| **CRP (mg/mL)** |  |  |  |
| n | 18^ǂ^ | 14 | 4 |
| Mean | 8.228 | 9.621 | 3.350 |
| SD | 9.9392 | 10.8626 | 2.7731 |
| Median | 5.000 | 5.000 | 3.000 |
| Min, Max | 0.10, 31.60 | 0.10, 31.60 | 1.00, 6.40 |
| **ESR (mm/hr)** |  |  |  |
| n | 38^ǂ^ | 21 | 17 |
| Mean | 10.9 | 13.3 | 7.9 |
| SD | 9.00 | 10.82 | 4.88 |
| Median | 8.5 | 8.0 | 8.0 |
| Min, Max | 1, 42 | 2, 42 | 1, 20 |
| **Physician's global assessment of disease activity** |  |  |  |
| n | 44 | 25 | 19 |
| Mean | 2.58 | 2.86 | 2.21 |
| SD | 2.348 | 2.405 | 2.281 |
| Median | 2.00 | 2.00 | 1.00 |
| Min, Max | 0.0, 8.0 | 0.0, 8.0 | 0.0, 7.0 |
| **CHAQ global assessment of well being** |  |  |  |
| n | 44 | 25 | 19 |
| Mean | 3.43 | 3.74 | 3.03 |
| SD | 3.005 | 3.218 | 2.731 |
| Median | 3.00 | 3.00 | 2.50 |
| Min, Max | 0.0, 10.0 | 0.0, 10.0 | 0.0, 8.5 |
| **CHAQ disability index score** |  |  |  |
| n | 42 | 25 | 17 |
| Mean | 0.506 | 0.590 | 0.382 |
| SD | 0.525 | 0.570 | 0.439 |
| Median | 0.313 | 0.250 | 0.250 |
| Min, Max | 0.00, 1.75 | 0.00, 1.75 | 0.00, 1.50 |
| **Number of Joints with Active Arthritis** |  |  |  |
| n | 44 | 25 | 19 |
| Mean | 3.1 | 3.9 | 1.9 |
| SD | 7.39 | 9.27 | 3.69 |
| Median | 1.0 | 1.0 | 1.0 |
| Min, Max | 0, 46 | 0, 46 | 0, 16 |
| **Number of Joints with Limited Range of Motion** |  |  |  |
| n | 44 | 25 | 19 |
| Mean | 1.7 | 2.7 | 0.4 |
| SD | 6.99 | 9.20 | 0.84 |
| Median | 0.0 | 0.0 | 0.0 |
| Min, Max | 0, 46 | 0, 46 | 0, 3 |

p>0.05 using Wilcoxon rank-sum test comparisons between patients on co-therapy and those with NSAID as primary therapy in the baseline disease measures

^ǂ^Includes all baseline data available regardless of whether the measure was continued at post-baseline visits during the study.

#### REFERENCES

1. The fourth report on the diagnosis, evaluation, and treatment of high blood pressure in children and adolescents. Pediatrics 2004 Aug;114(2 Suppl 4th Report):555-76.

2. Cassidy J, Kivlin J, Lindsley C, Nocton J. Ophthalmologic examinations in children with juvenile rheumatoid arthritis. Pediatrics. 2006;117(5):1843-5.
